# Baseline elevated Lp-PLA2 is associated with increased risk for re-stenosis after stent placement

**DOI:** 10.1186/1476-511X-13-41

**Published:** 2014-03-01

**Authors:** Dongdan Zheng, FanFang Zeng, Anping Cai, Huocheng Liao, Ling Liu, Ruofeng Qiu, Rulin Xu, Chun Xiao, Weiyi Mai

**Affiliations:** 1 Department of Cardiology, The First Affiliated Hospital of Sun Yat-sen University, 58 Zhongshan Road 2, Guangzhou 510080, China; 2 Department of Cardiology, Shenzhen Hospital of HongKong University, Shenzhen 518000, China; 3 Department of Cardiology, Guangdong Cardiovascular Institute, Guangdong General Hospital, Guangdong Academy of Medical Sciences, Guangzhou 510080, China; 4 Department of Cardiology, The 3rd People’s Hospital, Huizhou 516000, China

**Keywords:** Lipoprotein associated phospholipase A2, Coronary artery disease, Percutaneous coronary intervention

## Abstract

**Background:**

Lipoprotein associated phospholipase A2 (Lp-PLA2) is a novel biomarker for cardiovascular risk prediction. Whether increased Lp-PLA2 level is associated with re-stenosis after stent-placement is unclear.

**Methods:**

Totally 326 participants eligible for stent-placement were enrolled and divided into two groups according to baseline Lp-PLA2 levels (named normal and elevated groups). Baseline characteristics and clinical outcomes were compared between normal and elevated groups. The relationships between Lp-PLA2 and other risk factors with re-stenosis were evaluated.

**Results:**

Only the between-group difference of Lp-PLA2 was significant (123.2 ± 33.6 ng/mL vs 336.8 ± 85.4 ng/mL, P < 0.001) while other demographic and clinical characteristics between these two groups were comparable. Approximately 55.1% and 58.5% of participants in normal and elevated groups presented with acute coronary syndrome, and the percentage of tri-vessels stenoses was significantly higher in elevated group (40.8% vs 32.1%, P = 0.016). Nearly 96.0% and 94.0% of participants in normal and elevated Lp-PLA2 groups were placed with drug-eluting stents, and the others were with bare-metal stents. After 1 year’s follow-up, the incidence of clinical end-points was comparable (13.3% vs 15.4%, P = 0.172). Nevertheless, the incidence of re-stenosis was marginally higher in elevated Lp-PLA2 group (8.5% versus 4.6%, P = 0.047). With multivariate analysis, after adjustment for other risk factors, Lp-PLA2 remained an independent predictor for re-stenosis with a hazard ratio of 1.140. No synergistic effect between Lp-PLA2 and other risk factors for re-stenosis was found.

**Conclusion:**

Increased Lp-PLA2 level is associated with an increased risk of re-stenosis. Lp-PLA2 assessment may be useful in predicting subjects who are at increased risk for re-stenosis.

## Introduction

Atherosclerotic cardiovascular diseases (CAD), in terms of coronary heart disease (CHD), ischemic stroke and peripheral vascular disease, are still the leading causes of morbidity and mortality worldwide [1,2]. Currently, it has been well documented that inflammatory reaction plays critical roles on the initiation and progression of atherosclerosis and CHD [3,4]. Lipoprotein associated phospholipase A2 (Lp-PLA2), an enzyme excreted by inflammatory cells such as macrophages, nowadays has raised many concerns owing to its highly sensitive and specific features for vascular inflammation [5,6]. Previously, some basic research show that Lp-PLA2 plays casual role on atherosclerosis and inhibiting Lp-PLA2 ameliorates vascular inflammation and deters atherosclerosis progression [7,8]. Furthermore, some epidemiological studies and meta-analyses also consistently show that increased plasma level of Lp-PLA2 is associated with increased risk of cardiovascular events [9-12]. In light of these findings, Lp-PLA2 has been recognized as a novel and important predictor for cardiovascular risk assessment [13,14].

Currently, percutaneous coronary intervention (PCI) is the preferred choice for patients with acute myocardial infarction or with significant coronary artery stenoses. Nevertheless, despite dual anti-platelet and intensive statins therapy, the incidence of re-stenosis after stent placement is still high [15,16]. Therefore, identifying a specific and sensitive biomarker for predicting re-stenosis is of paramount importance. Owing to the significant and unequivocal implications of Lp-PLA2 on the initiation and progression of atherosclerosis and CAD, we speculated that increased plasma level of Lp-PLA2 at baseline might contribute to re-stenosis in patients with stent placement. If finally corroborated, we believed that in the future lowering Lp-PLA2 level with its specific antagonist would definitely reduce the risk of re-stenosis and doubtless improve the outcomes of patient with CAD.

## Method

### Study population and protocol

Current study was started in September of 2011 and ended in September of 2012. All participants were without previous history of atherosclerotic cardiovascular diseases. After angiographic evaluation, patients diagnosed as significant CHD and eligible for stent-placement were enrolled after written informed consent was obtained. Baseline demographic and clinical characteristics including traditional risk factors and laboratory results were collected. All participants were followed-up for 1 year via outpatient visit or telephone contact for detecting the first episode of pre-specified clinical outcomes.

### Routine laboratory parameters and Lp-PLA2 measurements

Fasting blood samples were drawn by veni-puncture in the morning. Measurements of routine laboratory parameters in current study were consistent to previous study report unless otherwise mentioned [17]. Plasma level of Lp-PLA2 was detected with commercial Lp-PLA2-ELISA kit (PLAC test; supplied by dia Dexus Inc, South San Francisco, California). All the procedures were performed according to the manufactures’ instructions. Based on previous recommendation [18], plasma Lp-PLA2 level less than 200 ng/mL is defined as within normal range, while equal or higher than 200 ng/mL is recognized as elevation. All participants were divided into two groups on the basis of the plasma level of Lp-PLA2.

### Clinical outcomes assessments post-stent placement

Clinical outcomes were defined as cardiovascular related death, nonfatal myocardial infarction, nonfatal ischemic stroke, and re-stenosis (> 50% stenosis of target lesion) by angiographic examination. Comparisons of clinical outcomes were conducted between groups with normal or elevated plasma levels of Lp-PLA2 during the 1 year’s follow-up. Risk factors for re-stenosis after 1 year’s follow-up were also evaluated.

### Statistical analysis

Continuous variable was presented as mean ± SD or median appropriately, and compared by the Student’s t-test when data was normally distributed, otherwise compared by Wilcoxon rank-sum test. Categorical data was showed as percentage and compared by χ^2^ test. Univariate and multivariate Cox proportional hazard regression were used to evaluate the associations between baseline demographic and clinical characteristics and incidence of re-stenosis after 1 year’s follow-up. Variables with p values < 0.10 were entered into multivariable model and variables with p values < 0.10 were stayed in the model. Synergistic effects between other risk factors and Lp-PLA2 for re-stenosis were also evaluated. Statistical analyses were performed by using SPSS software version 16.0 (SPSS, Inc., Chicago, Illinois). A value of p <0.05 was considered significant.

## Results

### Baseline demographic and clinical characteristics of participants

A total of 326 participants were enrolled in our current study, and accordingly, were divided into two groups on the basis of baseline plasma levels of Lp-PLA2. As presented in Table 1, only the difference of plasma Lp-PLA2 level between the normal and elevated groups achieved statistical significance. Male patients were predominant and accounted for 66.3% and 65.4% in each group, respectively. More than 50.0% of patients in each group have a history of hypertension. Each group had nearly 20% of patients with diabetes mellitus, and 29 (85.3%) patients in normal Lp-PLA2 group and 20 (83.3%) patients in elevated Lp-PLA2 group were treated with insulin and the rest (14.7% vs 16.7%, P = 0.283) were treated with sulfonylureas. More than 40.0% of participants in both groups have a family history of atherosclerotic cardiovascular diseases. Notably, in both groups, baseline levels of Hs-CRP were increased, and no significant differences were found across other risk factors such as total cholesterol, LDL-C, HDL-C and Lp(a) levels in both groups.

**Table 1:** Baseline characteristics of participants

**Variables**	**Normal Lp-PLA2 (n = 196)**	**Elevated Lp-PLA2 (n = 130)**	**P value**
Age (years)	57.4 ± 11.6	58.3 ± 13.2	0.115
Male (%)	130 (66.3)	85 (65.4)	0.172
Ever smoking (%)	103 (52.6)	71 (54.6)	0.168
HTN (%)	105 (53.6)	72 (55.4)	0.138
DM (%)	40 (20.4)	28 (21.5)	0.144
Family history (%)	82 (41.8)	58 (44.6)	0.126
BMI (Kg/m^2^)	25.4 ± 2.7	25.6 ± 2.2	0.278
CHOL (mmol/L)	4.64 ± 1.08	4.78 ± 1.12	0.389
LDL-C (mmol/L)	2.46 ± 0.79	2.68 ± 0.93	0.175
HDL-C (mmol/L)	1.08 ± 0.36	1.07 ± 0.33	0.405
Log TG (mmol/L)	0.17 ± 0.22	0.15 ± 0.21	0.141
Lp(a) (mg/dL)	27.6 ± 8.4	32.4 ± 10.5	0.062
CREA (umol/L)	93.5 ± 36.4	97.4 ± 32.8	0.804
BUN (mmol/L)	6.7 ± 1.3	7.1 ± 1.6	0.628
Uric acid (umol/L)	394.5 ± 122.5	377.6 ± 112.8	0.767
Hs-CRP (mg/L)	4.11 ± 0.92	4.07 ± 0.86	0.276
Lp-PLA2 (ng/mL)	123.2 ± 33.6	336.8 ± 85.4	<0.001
LVEF (%)	65.4 ± 12.6	63.8 ± 10.2	0.446
FS (%)	28.5 ± 3.3	27.2 ± 3.6	0.308
Anti-DM (%)	34 (85.0)	24 (85.7)	0.147
Anti-HTN (%)	98 (93.3)	67 (93.1)	0.325

Denote: HTN = hypertension, DM = diabetes mellitus, BMI = body mass index, CHOL = total cholesterol, TG = triglyceride, Lp(a) = lipoprotein(a), CREA = creatinine, BUN = blood uria nitrogen, LVEF = left ventricular ejection fraction, FS = fraction shortening.

### Angiographic characteristics and clinical presentations of participants

As shown in Table 2, participants in Lp-PLA2 elevated group more frequently presented with emergency conditions such as unstable angina and acute myocardial infarction than the normal Lp-PLA2 group (58.5% vs 55.1%). Moreover, the percentage of three vessels stenoses was higher in elevated Lp-PLA2 group than normal Lp-PLA2 group (40.8% vs 32.1%).

**Table 2:** Angiographic characteristics and clinical presentations of participants

**Variables**	**Normal Lp-PLA2 (n = 196)**	**Elevated Lp-PLA2 (n = 130)**	**P value**
UA (%)	75(38.3)	56(43.1)	0.042
AMI (%)	33(16.8)	20(15.4)	0.177
SA (%)	88(44.9)	54(41.5)	0.068
Tri-vessels (%)	63(32.1)	53(40.8)	0.016
Left main (%)	38(19.4)	24(18.5)	0.183

Denote: UA = unstable angina, AMI = acute myocardial infarction, SA = stable angina.

### Clinical outcome evaluation after 1 year’s follow-up

Approximately 96.0% (188) and 94.0% of participants (122) in normal and elevated Lp-PLA2 groups were placed with drug-eluting stents (DES), and the others were with bare-metal stents (BMS). During 1 year’s follow-up, all participants were adherent to dual anti-platelet (aspirin plus clopidogrel) and statins (atorvastatin or rosuvastatin) therapies. Other medications such as beta-receptor blockers, angiotensin-converting enzyme (ACE) inhibitor or angiotensin receptor blocker, and medications for hypertension or glucose control were prescribed accordingly, and no between-group differences were found. The incidence of clinical outcomes was comparable between normal and elevated Lp-PLA2 groups (13.3% versus 15.4%, P = 0.172, see Table 3). Moreover, each pre-specified outcome was further evaluated and only the incidence of re-stenosis was slightly but significantly higher in elevated Lp-PLA2 group than that of normal Lp-PLA2 group (8.5% vs 4.6%, P = 0.047).

**Table 3:** Clinical outcomes evaluation after 1 year’s follow-up

**Variables**	**Normal Lp-PLA2 (n = 196)**	**Elevated Lp-PLA2 (n = 130)**	**P value**
Cardiovascular related death (%)	6 (3.1)	3 (2.3)	0.138
Nonfatal MI (%)	5 (2.6)	3 (2.3)	0.216
Nonfatal Stroke (%)	6 (3.1)	3 (2.3)	0.138
Re-stenosis (%)	9 (4.6)	11 (8.5)	0.047
Total (%)	26 (13.3)	20 (15.4)	0.172

Denote: MI = myocardial infarction.

### Univariate and multivariate analysis for incidence of re-stenosis after 1 year’s follow-up and evaluation of synergistic effect

For further evaluating the value of each parameter for the prediction of re-stenosis after 1 year’s stent-placement, univariate and multivariate analyses were conducted. As showed in Table 4, several variables were found associated with re-stenosis after 1 year’s stent-placement with univariate analysis. However, with multivariate analysis, after adjustment for variables such as age, smoking, hypertension, total cholesterol, Lp(a), Hs-CRP, anti-platelet and medications for hypertension, only diabetes mellitus, LDL-C and Lp-PLA2 were found increasing hazard ratio for re-stenosis, whereas statins and medications for diabetes mellitus were found beneficial for reducing the incidence of re-stenosis (See Table 5). Synergistic effects between Lp-PLA2 and other risk factors for re-stenosis were evaluated by Cox proportional hazard regression and all values of P were higher than 0.05.

**Table 4:** Univariate analysis for re-stenosis

**Variables**	**Hazard ratio (95% CI)**	**P value**
Age	1.043 (0.906–1.083)	0.091
Male	1.052 (0.922–1.074)	0.106
Smoking	1.078 (1.003–1.125)	0.015
HTN	1.106 (1.018–1.276)	0.021
DM	1.145 (1.056–1.303)	0.007
Family history	1.008 (0.914–1.022)	0.108
BMI	1.002 (0.967–1.005)	0.455
CHOL	1.089 (0.953–1.117)	0.075
LDL-C	1.227 (1.104–1.420)	<0.001
HDL-C	0.895 (0.806–1.007)	0.303
TG	1.007 (0.926–1.011)	0.436
Lp (a)	1.103 (1.048–1.166)	0.014
CREA	1.012 (0.915–1.017)	0.344
BUN	1.005 (0.933–1.009)	0.428
Uric acid	1.017 (0.908–1.032)	0.447
Hs-CRP	1.168 (1.109–1.254)	<0.001
Lp-PLA2	1.233 (1.114–1.387)	<0.001
LVEF	1.017 (0.908–1.032)	0.160
FS	1.011 (0.933–1.019)	0.174
Anti-platelet	0.826 (0.801–0.944)	0.022
Statins	0.778 (0.723–0.806)	<0.001
Medications for HTN	0.909 (0.886–1.005)	0.096
Medications for DM	0.845 (0.817–0.896)	0.018

Denote: CI = confidence interval.

**Table 5:** Multivariate analysis for re-stenosis

**Variables**	**Hazard ratio (95% CI)**	**P value**
DM	1.078 (1.014–1.117)	0.023
LDL-C	1.165 (1.074–1.205)	0.008
Lp-PLA2	1.140 (1.068–1.195)	0.017
Statins	0.806 (0.788–0.865)	0.032
Medications for DM	0.837 (0.802–0.925)	0.037

## Discussion

Importantly, our current study shows that increased plasma level of Lp-PLA2 at baseline is associated with an increased risk for re-stenosis after 1 year’s stent placement. Moreover, patients with increased Lp-PLA2 level more frequently present with urgent situations such as unstable angina and acute myocardial infarction, which is consistent with previous findings that higher Lp-PLA2 level is strongly associated with a higher incidence of plaque rupture and major adverse events [19,20]. Additionally, our study also reveals that although a vast majority of patients present with Hs-CRP elevation, increased Hs-CRP level at baseline is not associated with re-stenosis after adjustment for other risk factors, and no synergistic effects between Lp-PLA2 and other risk factors for re-stenosis is found in current study.

Notably, in the past decades, the outcomes of patients with acute coronary syndrome have been significantly improved by stent placement, and with its excellent features of less invasive and highly efficient, PCI has become the principal therapy for patients with significant CHD [21-23]. However, the incidence of re-stenosis after PCI is still high in spite of prolongation of dual anti-platelet therapy and intensive statin treatment [15,24]. Many mechanisms have been identified responsible for this phenomenon. For example, endothelial dysfunction and activation, smooth muscle cells migration and proliferation, as well as continuous vascular inflammation have been demonstrated that individually and/or simultaneously contribute to in-stent re-stenosis [25,26].

Accordingly, Lp-PLA2 is an enzyme predominantly produced by leukocytes within atherosclerotic plaques and then excretes into circulation system [27,28]. It has now been well documented that plasma level of Lp-PLA2 is highly correlated with the concentration of Lp-PLA2 within the plaques [29]. Therefore, evaluating plasma level of Lp-PLA2 could accurately and promptly reflect the severity of vascular inflammation. Owning to its value of adding prognostic information to traditional risk evaluation algorithm, measurement of Lp-PLA2 has been recommended to patients with moderate or high cardiovascular risk [18,30]. However, whether increased plasma level of Lp-PLA2 is associated with an increased risk of re-stenosis after stent-placement is still unclear. For example, the study conducted by Moldoveanu and colleagues showed that there was no significant difference of baseline level of Lp-PLA2 in patients with and without re-stenosis after PCI [31]. Nevertheless, the result in our current study indicated that increased baseline level of Lp-PLA2 portended an increased risk of re-stenosis after 1 year’s follow-up. To our best knowledge, there might be some mechanisms contributing to this discrepancy. In the first place, the protocols of these two studies are quite different. In the study conducted by Moldoveanu et al., they divided patients into two groups according to the end-point in terms of with or without in-stent re-stenosis. While in our study, patients were assigned into normal and elevated Lp-PLA2 groups according to their baseline levels. Secondary, the duration of follow-up in the former study was only 6 months, while the patients in our study were followed-up for 1 year. Thirdly, the smaller sample size of Moldoveanu’s study might also be a potential reason for the negative finding. In light of the mechanisms associated with re-stenosis as mentioned above, we believed that the complex roles Lp-PLA2 plays on vessel walls might in part explain our findings. Pathophysiologically, after engulfed by macrophages, oxidative-LDL would be degraded into two major substrates, named lyso-phosphotidylcholine (Lyso-PC) and oxidized non-esterified fatty acids (oxNEFAs), by Lp-PLA2. Accordingly, Lyso-PC and oxNEFAs have potent effects in recruiting inflammatory cells, promoting platelets activation and aggregation, impairing endothelial functions as well as enhancing smooth muscle cells proliferation and migration which subsequently lead to neointima formation and re-stenosis [32-34]. Taken together, it was reasonable to speculate that increased plasma level of Lp-PLA2 was associated with higher risk of re-stenosis. Nevertheless, the study conducted by Turunen and colleagues showed that local adenovirus-mediated Lp-PLA2 gene transfer resulted in a significant reduction in neointima formation in balloon-denuded rabbit aorta [35]. However, the pathophysiology of aorta denudation by balloon was somewhat, if not completely, different from that of vascular inflammation inducing by hyperlipidemia and endothelial dysfunction and activation. Furthermore, a large number of clinical studies have consistently demonstrated the positive relationship between increased plasma level of Lp-PLA2 and adverse clinical outcomes [8,9,11,20]. Additionally, as stent placement may stimulate local inflammatory reaction and up-regulate inflammatory cytokines such as tumor necrosis factor-alpha expression, which in turn leads to platelet activating factor and Lp-PLA2 correspondingly increase. However, since only baseline Lp-PLA2 level has been measured, our study was impossible to make any conclusion that whether the change of platelet activating factor and Lp-PLA2 after stent-placement would also have significant effects on re-stenosis. Furthermore, since the percentages of drug-eluting stent and bare-metal stent placement in the normal and elevated groups were comparable, any bias related to stent-placement might also be excluded. In the future, conducting a prospective research to investigate whether reducing Lp-PLA2 level could decrease the risk of re-stenosis would definitely be a milestone with regard to the new era of CAD therapy.

On the other hand, at the very beginning, Lp-PLA2 was considered as a cardio-protective factor due to its effects on degrading platelet activating factor, a potent substrate in enhancing vascular inflammation and impairing cardiac function [36]. Nevertheless, since the positive relationship between Lp-PLA2 level and increased CVD risk has been firstly reported in WOSCOPS study in 2000 [37], many studies thereafter have consistently corroborated the detrimental effects of Lp-PLA2 on cardiovascular system [8,38,39]. Currently, the results from our study also indicated that patient with increased Lp-PLA2 level appeared to present more severe and urgent condition than that with normal Lp-PLA2 level, which might be partially explained by the adverse effects (expanding necrotic lipid core and thinning fibrous cap) Lp-PLA2 imposes on vessel walls [8,40]. The higher percentage of patients with three vessels stenoses in elevated Lp-PLA2 group might also partially reflect the adverse effects Lp-PLA2 exerts on atherogenesis and progression.

Thirdly, due to the production of inflammatory cytokines such as interleukin-6 tumor necrosis factor-alpha and platelet activating factor from adipose tissues, obesity is considered as one of the major risk factor for cardiovascular events such as in-stent re-stenosis [41,42]. Interestingly, previous one study showed that increased Lp-PLA2 might compensate for the adiposity-associated increases in inflammatory and oxidative burden [43]. Therefore, it might be reasonable to speculate that in patients with obesity, the incidence of re-stenosis in higher Lp-PLA2 level group might be lower than the lower Lp-PLA2 group. However, in our current study, no significant difference of incidence of re-stenosis was found in obese patients with normal and increased Lp-PLA2 level (33.3% vs 36.4%, P = 0.208).

Finally, since a vast majority of participants in current study were with increased level of Hs-CRP (>3 mg/L), we therefore further evaluated the relationship between Hs-CRP level and re-stenosis. With univariate analysis, Hs-CRP appeared to be a potential predictor for re-stenosis. Nevertheless, after adjustment for other risk factors, Hs-CRP did not remain independently associated with re-stenosis, which was consistent with previous studies [44,45], in which increased Hs-CRP level before PCI were not associated with in-stent re-stenosis. We believed that this negative finding might be partially due to the unspecific characteristic of Hs-CRP, with respect to Hs-CRP elevation could be confounded by other diseases such as latent and modest infection [46,47]. Contrary to Hs-CRP, Lp-PLA2 still remained an independent predictor for re-stenosis after adjustment for other risk factors (hazard ratio: 1.140, 95% confidence interval: 1.068-1.195), which we believed was predominantly due to its high specificity for vascular inflammation in the setting of atherosclerotic cardiovascular diseases [6,14]. In order to explore any synergistic effect between Lp-PLA2 and other risk factors for re-stenosis, Cox proportional hazard regression were performed and no significant synergism was found which we considered might be partially ascribed to the relatively small sample size of current study and future study is warranted to further investigate whether any synergistic effect exists between traditional risk factor and Lp-PLA2 for re-stenosis after stent-placement.

## Conclusion

The results of our study indicate that increased plasma level of Lp-PLA2 at baseline is associated with increased risk of re-stenosis after 1 year’s stent-placement, and measurement of Lp-PLA2 may be helpful and useful for identifying subjects who are at an increased risk for re-stenosis. Future study is warranted to investigate whether decreasing plasma level of Lp-PLA2 with specific antagonist will reduce the incidence of re-stenosis.

## Competing interests

The authors declare that they have no competing interests.

## Authors’ contributions

DZ, FZ, HL and LL predominantly performed this study, RQ and RX helped to collect the demographic and clinical characteristics of all participants, HL and AC wrote this article, and CX and WM designed this study and WM helped to revise final version of current manuscript. All authors read and approved the final manuscript.

## Authors’ information

Dongdan Zheng, FanFang Zeng, Anping Cai: co-first authors.
